# Alpha7 acetylcholine receptor autoantibodies are rare in sera of patients diagnosed with schizophrenia or bipolar disorder

**DOI:** 10.1371/journal.pone.0208412

**Published:** 2018-12-06

**Authors:** Carolin Hoffmann, Jo Stevens, Shenghua Zong, Daan van Kruining, Abhishek Saxena, Cem İsmail Küçükali, Erdem Tüzün, Nazlı Yalçınkaya, Marc De Hert, Emiliano González-Vioque, Celso Arango, Jon Lindstrom, Marc H. De Baets, Bart P. F. Rutten, Jim van Os, Peter Molenaar, Mario Losen, Pilar Martinez-Martinez

**Affiliations:** 1 Department of Psychiatry and Neuropsychology, School for Mental Health and Neuroscience, Maastricht University, Maastricht, the Netherlands; 2 Department of Neuroscience, Institute for Experimental Medical Research (DETAE), Istanbul University, Istanbul, Turkey; 3 University Psychiatric Centre Catholic University Leuven, Campus Kortenberg, Kortenberg, Belgium, Department of Neurosciences KU Leuven, Belgium; 4 Child and Adolescent Psychiatry Department, Hospital General Universitario Gregorio Marañón, School of Medicine, Universidad Complutense, IiSGM, CIBERSAM, Madrid, Spain; 5 Department of Neuroscience, University of Pennsylvania Perelman School of Medicine, Philadelphia, Pennsylvania, United States of America; University of Sydney, AUSTRALIA

## Abstract

The α7 acetylcholine receptor (AChR) has been linked with the onset of psychotic symptoms and we hypothesized therefore that it might also be an autoimmune target. Here, we describe a new radioimmunoassay (RIA) using iodine 125-labelled α-bungarotoxin and membrane extract from transfected HEK293 cells expressing human α7 AChR. This RIA was used to analyze sera pertaining to a cohort of 711 subjects, comprising 368 patients diagnosed with schizophrenia spectrum disorders, 140 with bipolar disorder, 58 individuals diagnosed of other mental disorders, and 118 healthy comparison subjects. We identified one patient whose serum tested positive although with very low levels (0.2 nM) for α7 AChR-specific antibodies by RIA. Three out of 711 sera contained antibodies against iodine 125-labelled α-bungarotoxin, because they precipitated with it in the absence of α7 AChR. This first evidence suggests that autoantibodies against α7 AChR are absent or very rare in these clinical groups.

## Introduction

Recently, autoantibodies to neuronal cell surface antigens have been identified in patients with psychotic disorders [1]. The alpha7 nicotinic acetylcholine receptor (α7 AChR) represents an interesting target which has received little attention in this respect. It is an ion channel involved in auditory gating; disturbed signaling of this channel can lead to auditory hallucinations, one of the prominent symptoms in psychotic disorders such as schizophrenia and bipolar disorders [2]. The α7 AChR-encoding gene, CHRNA7, is a susceptibility candidate gene in schizophrenia and the α7 AChR protein is currently seen as one of the most promising drug targets for schizophrenia [3]. While mRNA expression levels of α7 AChR were unaffected [4], protein expression levels were reduced in post mortem neuronal tissue of patients diagnosed with schizophrenia [5–7]. Taken together, this led us to the hypothesis that autoantibodies could reduce α7 AChR protein levels and thereby contribute to psychotic disorders in a subgroup of patients. To our knowledge, only one study has investigated the presence of such antibodies in psychotic disorders: in 2009, Chandley and colleagues reported elevated α7 AChR autoantibodies in schizophrenia patients (23% of n = 21) as compared to controls (0% of n = 17), measured by enzyme-linked immunosorbent assay [8]. Another study reported that elevated blood plasma levels of (1–208) α7 AChR-specific antibodies are a possible risk-factor for early-onset Alzheimer’s disease [9, 10]. This is reminiscent of the reported similarity between α7 AChR dysfunction in Alzheimer’s disease and schizophrenia-spectrum disorders [11].

In the autoimmune disease myasthenia gravis (MG), where autoantibodies against the α1 AChR damage the neuromuscular junction resulting in muscle weakness, a radio-immunoprecipitation assay (RIA) has been proven to be a highly specific and sensitive diagnostic tool [12–14]. It uses radioactively (iodine, ^125^I) labeled α-bungarotoxin, a neurotoxin that binds with very high affinity to the muscle nicotinic AChR. The advantage of this assay is that it screens for antibodies against the whole transmembrane receptor, and therefore can also detect antibodies directed against conformational epitopes. A similar RIA is used for detecting autoantibodies targeting the ganglionic α3 AChR in autoimmune autonomic ganglionopathy (AAG) [15, 16].

We aimed to use this knowledge and adapt it to the α7 AChR, which has a very similar structure and also binds to α-bungarotoxin. The various nicotinic AChRs consist of five subunits that form an ion-channel [17, 18]. Their subunits combine in different variations, e.g. the α7 forms a homopentamer whereas the α1 AChRs of the adult neuromuscular junction consists of two α1 subunits, a β1, a δ and a ε subunit [17, 19, 20]. We adopted a novel RIA for α7 AChR that uses membrane extract from α7 AChR expressing human embryonic kidney (HEK) cells as antigen together with ^125^I-labelled α-bungarotoxin. In order to test the hypothesis that auto-antibodies against α7 AChR are more prevalent in patients with mental disorders than in controls, this RIA was used to screen serum samples of 368 patients diagnosed with schizophrenia spectrum disorders, 140 with bipolar disorder, 58 patients with other mental disorders (OMD), and 118 healthy controls.

## Methods

### Study population

Samples and patient data were collected according to national and institutional ethical guidelines and the Helsinki Declaration. Ethical approval was given by all responsible clinical research ethics committees: the medisch-ethische toetsingscommissie (METC) azM/UM, the ethische commissie onderzoek UZ / KU Leuven (EC onderzoek), Comité Ético de Investigación Clínica (CEIC) Hospital Gregorio Marañón and the Institutional Review Board of Istanbul Faculty of Medicine for Turkey. Written informed consent was given by all participants. The study population was composed of different neuropsychiatric cohorts and controls. It included sera of patients with a broad spectrum of psychotic disorders and healthy individuals from different locations within Europe. The capacity to give written informed consent was evaluated by a psychiatrist by a face-to-face interview using a series of open-ended questions evaluating comprehension, reasoning, choice making and appreciation skills of the patient. For participants under age 18, informed consent was obtained from their guardians/representatives.

In total we included 4 different cohorts:

Cohort 1 consisted of 245 patients with DSM-IV diagnosis of schizophrenia, schizoaffective disorder or bipolar disorder, 13 healthy individuals and 35 with other mental disorders (OMD). Persons were included between November 2003 and July 2007 at the University Psychiatric Center, Katholieke Universiteit Leuven, Kortenberg, Belgium. This cohort was previously analyzed for metabolic disturbances [21].
Cohort 2 consisted of individuals with DSM-IV diagnosis of schizophrenia that was selected from patients aged above 50 because previous publications hypothesized increased autoantibody prevalence in elderly individuals (Cohort 2a, N = 64) and bipolar disorder (Cohort 2b, N = 99). Sera were collected between 2011–2012 at the Istanbul University, Aziz Sancar Institute of Experimental Medicine.
Cohort 3 comprised of patients recruited from January 2007 to December 2010, at the Child and Adolescent Psychiatry Department of the Hospital General Gregorio Marañon. Patients had a diagnosis of a mental disorder according to DSM-IV criteria and were drug-naïve or with less than 10 days of second generation antipsychotic treatment at the time of serum sampling. This cohort also included 46 healthy controls that were recruited among patients’ friends, colleagues and neighbors. This cohort was previously described in the SLiM Study [22].
Cohort 4 were anonymized blood donors who underwent a general health screening; their sera were collected during March 2014 from Sanquin Maastricht (N = 105).

Table 1 summarizes the patients’ demographic characteristics.

**Table 1:** Demographic data of the studied cohorts.

		Total	Cohort 1	Cohort 2a	Cohort 2b	Cohort 3	Cohort 4
**N**		**634**	**293**	**64**	**99**	**73**	**105**
**Age**	**Mean**	33.4	28.5	57.8	40.0	21.7	42.9
	**(range)**	(16–80)	(16–58)	(51–80)	(19–74)	(8–75)	(19–59)
**Gender**	**Male N**	355	168	33	46	45	63
	**(%)**	(53.7)	(57.3)	(51.6)	(46.5)	(61.6)	(60)
** **	**Female N**	279	125	31	53	28	42
	**(%)**	(42.2)	(42.7)	(48.4)	(53.5)	(38.4)	(40)
**Diagnosis**	**Healthy N**	118	13	0	0	0	105
	**OMD N**	58	35	0	0	23	0
	**Psychotic disorders N**	458	245	64	99	50	0
	**Subdiagnoses**						
	schizophrenia	193	122	64	0	7	0
	schizoaffective	40	40	0	0	0	0
	brief psychotic	3	0	0	0	3	0
	FEP	43	43	0	0	0	0
	psychosis NOS	22	0	0	0	22	0
	delusional disorder	4	0	0	0	4	0
	substance induced	2	0	0	0	2	0
	bipolar disorder	148	40	0	99	9	0
	schizophreniform	3	0	0	0	3	0

OMD, other mental disorders; FEP, first episode psychosis; NOS, not otherwise specified

### Cell culture

We used HEK293 cells stably transfected with the human α7 AChR subunit and the chaperone RIC-3. The cloning and culture of the cell line was described in detail [23]. Cells were maintained in DMEM with penicillin (100 U/mL), streptomycin (100 mg/mL), and 10% fetal bovine serum. Zeocin (0.5 mg/mL) was used for selection of α7 AChR expression, and G418 (0.6 mg/mL; both from Invitrogen) was used for the selection of RIC-3 expression. In order to increase the expression of mature functional AChRs in HEK cells, we used 4-phenylbutyric acid (PBA) and sodium valproate (VPA), which act as chemical chaperones. Cells were grown with 1 mM VPA (P4543, Sigma) and 1.5 mM PBA (SC-200652, Santa Cruz) for 2 weeks before antigen was extracted.

### Antigen extraction

Cells from 225 cm^2^ culturing flasks were collected in 2 mL ice cold PBS by scraping and pipetting. The cell suspension was centrifuged at 1000 g for 5 min; the supernatant was then discarded and the pellet was stored at -80°C.

Extract was prepared by thawing the cell pellet of one 225 cm^2^ culturing flask, transferring the cells to a 1.5 mL centrifuge tube, adding 1 mL homogenization buffer [phosphate-buffered saline (PBS) supplemented with 1 M NaN_3_, 0.5 M ethylenediaminetetraacetic acid (EDTA), 0.01 M iodoacetamide, 0.1 M phenylmethylsulfonyl fluoride and protease inhibitor cocktail (11836 145 001, Roche; 1 tablet per 50 mL)] and homogenizing (Ultra-Turrax). The membrane fraction was pelleted by centrifugation for 15 minutes at 15000 g at 4°C. Then the supernatant was discarded and membrane components were solubilized in extraction buffer (homogenization buffer supplemented with 2% Triton-X100). The Ultra-Turrax was used to homogenize the extract followed by incubation on a shaker for 1 hour at room temperature. The samples were then centrifuged as before and the supernatant with membrane antigens was transferred to a new 1.5 mL centrifuge tube. The extract was diluted 1:10 in extraction buffer, homogenized and centrifuged as before. The supernatant was stored at 4°C and used within 48 hours.

### Radioimmunoassay for detection of α7 AChR antibodies

A monoclonal rat α7 AChR antibody (clone AB319, ab24644, Abcam) was used as positive control to establish and validate the assay. IgG concentration of the AB319 was determined by ELISA, as described [24]. Using its standard curve, the concentration of extract and radioactive α-bungarotoxin was diluted in different concentrations to gain the highest ratio between saturation-levels of coprecipitated ^125^I α-bungarotoxin and background signal. Each experiment included a standard curve in duplicate measures with AB319; serum samples were tested in triplicates. In each tube, 2.5 μL of human serum/ AB319, 11.34 fmol ^125^I-labelled α-bungarotoxin (NEX126050UC, Perkin Elmer), 12.5 μL membrane extract (as described above) and PBS was added to reach a total volume of 20 μL/condition. In the positive control the AB319 was used (instead of human serum) with additional 2.5 μL of normal rat serum. Sera were incubated with the antigen over night at room temperature to allow binding. The next morning, 100 μL goat-anti-rat IgG (undiluted serum from an immunized goat, Eurogentec, Liege) was added to the standard curve samples; for human serum samples 150 μL goat-anti-human IgG (undiluted serum from an immunized goat, Eurogentec) was used instead. The samples were incubated for 4 hours at room temperature to allow immune-complex formation. Samples were washed 3 times by adding 1 mL PBS with 2% (v/v) Triton-X100 and centrifugation for 5 minutes at 13000 RPM at 4°C. The radioactivity of the pellet was measured in counts per minute (CPM) using a 2470 Wizard2 gamma counter (Perkin Elmer, USA). Counts were corrected for the radioactive decay and efficiency of the counter. The cut-off for positivity was determined as the mean of healthy cohort plus 2 times the standard deviation.

### Cell-based assays

A cell-based assay for human α7 AChR was developed to confirm antigen expression and detect binding of human autoantibodies. Cells stably expressing α7 AChR were initially cultured for 5 days with chemical chaperones PBA and VPA, as aforementioned. 5 ml cells were then plated at a density of 200,000 cells/mL on poly-D-lysine-coated (#P7280, Sigma) cover glasses (diameter 12 mm, #ECN 631–1577, VWR) in cell culture dishes (diameter 60 mm, #628160, Greiner). In addition, transiently transfected cells were used. For this, HEK293 cells were grown on coverslips in cell culture dish for 24 h and then transfected with lipofectamine (11668–019, Invitrogen) and 4 μg (ratio 3 to 1) of the same plasmids used to establish the aforementioned stable cell line (expressing the human α7 AChR and the molecular chaperone RIC-3). Expression of the antigen was allowed for 2 days.

We used both transiently and stably transfected cells for two variants of the CBA termed ‘live CBA’ and ‘fixed CBA’. For the fixed CBA, cells were incubated in 3.5% formaldehyde (#87837.180, VWR) for 10 min and permeabilized with 0.3% Triton-X100 for 10 min. After blocking with 1% bovine serum albumin (BSA, A7906, Sigma) in PBS for 1 h, cells were incubated with human sera diluted 1:40 in PBS with 1% BSA together with AB319 (1:800) for 1 h at room temperature. Bound antibodies were detected by goat-anti-human-IgG Fcγ-Alexa488 (1:750, #109-546-170, Jackson Laboratories) and goat-anti-rat-594 (1:500, #112-585-143, Jackson Laboratories). For the live CBA, cells were first incubated with diluted patient serum for 1 h, washed with PBS, and then fixed in 3.5% formaldehyde. Cells were then permeabilized and in the case of transiently transfected cells additionally incubated with AB319. Bound antibodies were stained with secondary antibodies as above. Both for the live and the fixed CBA, expression of α7 AChR was tested in parallel with Alexa488-labelled α-bungarotoxin (1:1000, B13422, Molecular Probes). All cover glasses were mounted onto 7 μL DAPI mounting medium (#H-1200, Vector Laboratories). Stainings were evaluated by a blinded observer with extensive experience in scoring other CBAs that were established in our lab including the detection of autoantibodies to N-methyl-d-aspartate receptor, α-amino-3-hydroxy-5-methyl-4-isoxazolepropionic acid receptor, GABAB receptor, GABAA receptor, leucine-rich, glioma inactivated 1, contactin-associated protein-like 2, enzyme glutamate decarboxylase isotype 65 and 67. Briefly, a staining was considered positive when transfected, but not untransfected cells showed membrane reactivity by human serum. Images were taken at 14-bit intensity resolution with an XC10 camera on a BX51 microscope (both from Olympus) at exposure times of 500 ms, 1000 ms, and 50 ms for the red, green and blue channels respectively.

As a standard curve, we used a fixed CBA of transiently transfected cells and a dilution series of AB319 (dilution range from 1:100 to 1:25600 with a dilution factor 4). As a dilution buffer, 5% goat serum in PBS was used, which also served as negative control. For each dilution, two images were taken from a random location within the same slide of transfected cells at 40x magnification. Quantification of microphotographs was performed with ImageJ by firstly selecting the region of interest (ROI) based on the DAPI staining and measuring the mean fluorescent intensity (MFI, ranging from 0–16383) in each ROI.

### Statistical analysis

To test for the differences of α7 AChR antibody presence in the different cohorts, we performed a Kruskal-Wallis test and a Chi-Square test for categorical values (positive/negative antibody test results). Further, we performed a Pearson’s correlation for statistical tests in which both variables were continuous (that is, age and autoantibody concentration) and a t-test to test whether or not the mean differed between RIA measurements with and without α7 AChR extract. All analyses were done in IBM SPSS Statistics version 23.0 for Windows or GraphPad Prism 6.

## Results

**Fig 1:**
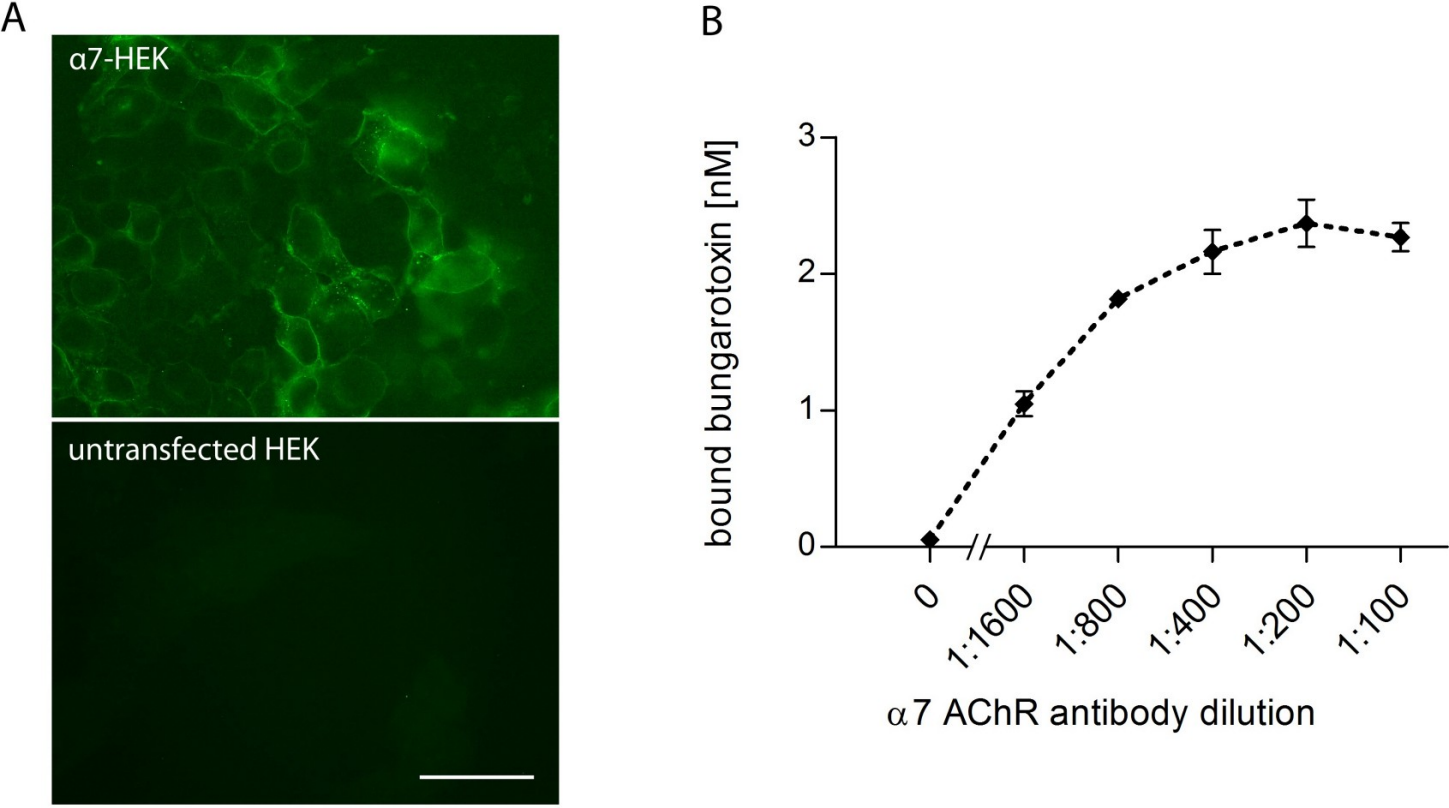
Analysis of a7 AChR expression in HEK293 cells and antibody binding to a7 AChR in cell membrane extracts. (A) α-bungarotoxin-Alexa488 staining of α7 acetylcholine receptor (AChR) stable transfected, fixed HEK293 cells (top panel) and untransfected, fixed HEK293 cells (lower panel). The scale bar is 50 μm. (B) Standard curve with α7 AChR antibody (AB319) in a RIA. The RIA was performed with ^125^I α-bungarotoxin and membrane extracts of α7 AChR transfected HEK293 cells. Data points represent the mean of triplicates and error bars indicate the standard deviation.

Immunofluorescent staining of stably transfected HEK cells that had been cultured in the presence of chemical chaperones showed expression of α7 AChR (as detected with α-bungarotoxin-Alexa488, Fig 1A). Based on this, we attempted to develop a CBA for detection of α7 AChR antibodies, in analogy to other CBAs that we use to detect autoantibodies in sera of patients with autoimmune encephalitis (e.g. against the N-methyl-D-aspartate receptor [25]). However, the culturing with chemical chaperones led to cell clustering and high background of the immunofluorescent staining when using sera (see Part C of S1 Fig for details). After transient transfection, α7 AChR protein expression was much higher in individual cells as detected by the monoclonal α7 AChR antibody AB319, which recognizes a conformational and non-conformational intracellular epitope. A standard curve of AB319 binding on transiently transfected and permeabilized HEK293 cells is represented in S2 Fig However, most of the expressed α7 AChR remained intracellular (as detected with AB319 in permeabilized cells) and did not effectively reach the plasma membrane (as detected with fluorescently-labelled α-bungarotoxin in non-permeabilized cells), see part A and B of S3 Fig. To overcome these technical limitations, we cultured stably transfected HEK cells in the presence of chemical chaperones for 2 weeks, prepared membrane extracts from these cells and then specifically labelled the α7 AChR within these extracts with ^125^I α-bungarotoxin. These labelled extracts were then used to analyze serum samples for the presence of antibodies against the α7 AChR by radioimmunoprecipitation (RIA). The functionality of the RIA was confirmed by precipitating ^125^I α-bungarotoxin with serial dilutions of the monoclonal α7 AChR antibody AB319. A representative standard curve with titrations of the rat monoclonal α7 AChR antibody is shown in Fig 1B with a background around 0.05 nM (~10 cpm) of bound α-bungarotoxin and a saturation 2.27 nM (~1000 cpm); the maximum available counts per condition were ~10,000 cpm (24 nM). Based on the lowest concentration of AB319 that gave a clear positive signal in the RIA for α7 AChR (at 1:1600 dilution), we estimated that the detection limit of the RIA was below 0.2 nmol/L IgG (assuming that autoantibodies and AB319 have a similar affinity to the α7 AChR; the AB319 was measured by ELISA to have a IgG concentration of 0.05 g/L). This RIA was then used to screen sera for the presence of human α7 AChR autoantibodies.

### Antibody reactivity to α7 AChR RIA is rare

**Fig 2:**
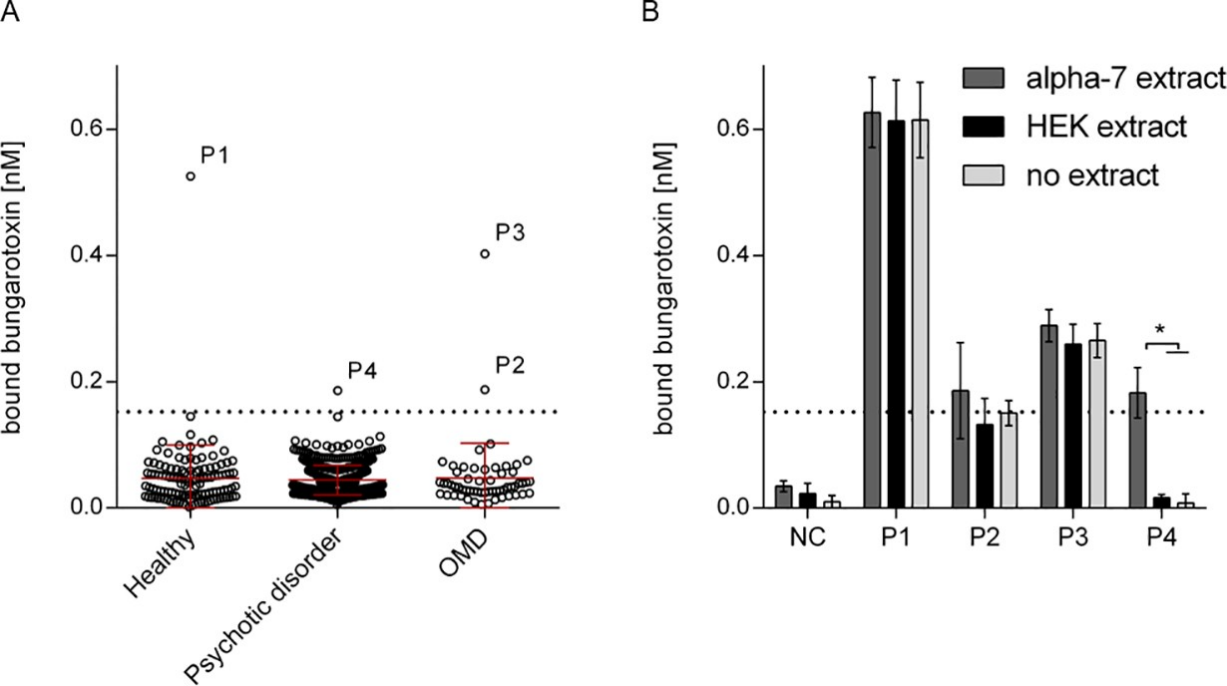
Radioimmunoassay for the detection of α7 AChR antibodies in human sera. (A) Tested sera were from healthy individuals, patients with psychotic disorders, and patients with other mental disorders (OMD). Results represent the nM α-bungarotoxin precipitated by human sera. Error bars indicate the standard deviation. The dotted line indicates the cut-of level, defined by the mean of the healthy cohort plus 2 times the standard deviation. (B) Positive sera (above the cut-off level in panel A) were retested in the radioimmunoassay for α7 AChR specificity, by performing the RIA with α7 AChR membrane extract, membrane extract without α7 AChR, and with lysis buffer only. These positive sera were named P1-P4 and a negative control (NC) was a serum sample with a consistently negative RIA result. Results are the means of triplicate measurements with standard deviation; * represents a p-value < 0.05 when performing multiple t-tests with Bonferroni correction.

By measuring antibody reactivity in the α7 AChR RIA, we found antibody reactivity in individuals from the different groups; i.e. one healthy subject, two patients diagnosed with OMD, and one patient diagnosed with psychotic disorders showed positive results (cut-off = mean of healthy titers+2*SD). To test the specificity of the results, the RIA was then repeated with different antigen extracts. These extracts were either (i) the same α7 AChR containing membrane extract, (ii) membrane extract without α7 AChR or (iii) lysis buffer without protein. All but one serum reacted similarly in the 3 different conditions, indicating that the antibodies were not specific to α7 AChR (Fig 2B indicates reactivity of sera in these 3 conditions). Because some sera precipitated the radiolabeled α-bungarotoxin in the absence of any added protein, antibodies were likely to bind directly to the α-bungarotoxin. Only one serum reacted specifically to α7 AChR; but the binding was low (specific IgG concentration equivalent to 0.2 nM of α-bungarotoxin). This level of binding was within the range of 3 SDs above average. Considering the number of tested sera (711), and the number of measurements expected to be in this range by chance (2.3%) this finding cannot be considered significant. However, this binding was consistently observed in several replications of the experiment. The serum was from a patient diagnosed with schizophrenia (Fig 2B, P4) from cohort 2a, a 55-year-old female patient with a chronic psychosis with disease duration of 20 years. She mostly had displayed positive symptoms of schizophrenia (delusions, hallucinations). The only reported comorbidity was hypertension. The sera with antibodies to (or cross-reacting with) ^125^I α-bungarotoxin (as seen in Fig 2B) were from a 40-year-old healthy male (P1, highest counts), a 17-year-old male with adjustment disorder (P2, cohort 3) and an 8-year-old male with attention deficit hyperactivity disorder (P3, ADHD, cohort 3). RIA results of the complete cohort are represented in the supporting information (S1 Table).

The relevant sera were also tested by live and fixed CBAs with transient transfection of the α7 AChR. None of these sera (including serum P4, which was positive with low titers for α7 AChR by RIA), were found positive in either CBA (part C of S3 Fig). The interpretation of the CBA results was difficult, however, being limited due to the very low α7 AChR expression in the cell membranes, as detected with fluorescently-labelled α-bungarotoxin in the live CBA (part B of S3 Fig).

## Discussion and conclusions

We identified one patient (P4) diagnosed with schizophrenia to be positive with low titers for α7 AChR antibodies and 3 individuals with antibodies against α-bungarotoxin. The positive patient would not be considered positive if the threshold was increased from mean+2SD to mean+3SD and might thus be a false-positive. Moreover, in a CBA with transiently transfected cells, we could not confirm the presence of α7 AChR antibodies in the serum of this patient. These results indicate that the prevalence of α7 AChR antibodies is very low and therefore we believe that the finding of the low positive α7 AChR antibody levels in one patient is not likely to be pathophysiologically relevant.

These results seem to be at odds with the findings by Chandley *et al*. which however might be explained by the differences in methodologies. It seems possible that the (1–208) α7 AChR peptide contains epitopes that are not accessible in the native form of the channel. This peptide was also used for testing sera of AD patients for the presence of α7 AChR antibodies [9, 10]. Using this method, sera from all individuals, including healthy controls, showed varying levels of α7 AChR antibodies and the authors suggested that these are the result of an immune reaction to respiratory (or other) infections because they could be related to the number and/or severity of infections an individual was exposed to during his/her life time. This could also explain their finding of increased prevalence of autoantibodies with age. In the development of our RIA, the reactivity of such bystander reactivity was likely reduced by the dilution of antigen to detect only higher affinity binding. A parallel testing of the same sera in the different assays (e.g. ELISA, CBA, and RIA) would be needed to determine the comparability between these methods. Further efforts in optimizing a cell-based assay for this antigen would also be beneficial. The immunoprecipitation assay used here requires further validation, since it has not previously been used for detecting CNS autoantibodies and its sensitivity rests entirely upon results with one weak positive patient sample and from a rat monoclonal (detected with a distinct secondary antibody that may differ in IgG affinity and quantum yield).

A possible limitation of our study is that we analyzed only the serum, whereas the combination of autoantibody testing in serum and cerebrospinal fluid (CSF) is advised for most autoimmune diseases of the central nervous system, because in some cases only one of the two is tested positive [26, 27]. However, CSF examination is not yet among the routine diagnostic procedures in psychiatry and CSF samples are therefore difficult to obtain [28].

Another consideration is that α-bungarotoxin might compete with the autoantibodies on binding to the α7 AChR. The autoantibodies in AChR-MG predominantly bind to the extracellular domain of the α1 subunits adjacent to the neighboring ε subunit, meaning that each receptor has two antibody binding sites [29]. In the homomeric α7 AChR, with five identical subunits, antibodies could potentially bind to all five subunits. Also, α-bungarotoxin binds to the C-loop of all five subunits with high affinity [30]. Thus, if antibody binding competes with α-bungarotoxin, the reactivity in RIA would be reduced or even abolished.

A complication of the RIA was the unexpected high frequency of reactivity to ^125^I-α-bungarotoxin among the tested sera (3 out of 711; 0.4%). Since the same radioligand is also used for the diagnosis of α1 AChR antibodies in MG, this outcome implies the possibility of false positive results for this assay. A large study from a diagnostic laboratory and reference center for MG reported that out of 4018 sera positive by RIA with ^125^I-α-bungarotoxin-labelled AChR, two sera (0.05%) had antibodies to the radiolabel only [14]. It was therefore recommended to confirm apparently positive results by retesting sera with ^125^I labelled α-bungarotoxin in the absence of muscle AChR [11, 31]. It has been reported earlier that patients, who were treated with anti-snake venom (from *Equus caballus*) or experimental snake venom therapy, demonstrate high false positive titers of anti-AChR Ab in their serum as measured by RIA [32–34]. It is possible that these individuals had been exposed to α-bungarotoxin-like antigens.

Although we did not identify patients with high titers of α7 AChR autoantibodies in the patient groups tested here, we believe that the different α7 AChR autoantibody detection-methods might be useful in other neurological diseases with cholinergic dysfunction, e.g. Alzheimer’s disease or autoimmune encephalitis. Given the paucity of positive patient samples both the RIA and the CBA for detecting α7 AChR autoantibodies will require further validation.

## Supporting information

S1 Fig Cell based assay with stable transfected cells for detection of α7-AChR autoantibodies. Cells stably expressing α7 AChR were cultured with chemical chaperones. α7 AChR expression was detected by fixed CBA with AB319 followed by secondary goat-anti-rat-Alexa594 (A, red fluorescence) or by live CBA with Alexa488-labelled α-bungarotoxin (B, green fluorescence). C) represents a staining with serum (diluted 1:40) from a healthy control on live CBA. Bound antibodies were detected by goat-anti-human-IgG Fcγ-Alexa488. The scale bar represents 50 μm. Cell nuclei are stained with DAPI (blue fluorescence).
(TIF)

S2 Fig α7-AChR autoantibody (AB319) staining using fixed cell based assay with transiently transfected cells. **A)** AB319 (at dilutions as indicated above the images) was used to stain paraformaldehyde-fixed, Triton-X100-permeabilized, α7 AChR-expressing HEK293 cells after transient transfection. AB319-binding was detected by goat-anti-rat IgG-Alexa594 (1:500; red fluorescence). Cell nuclei are stained with DAPI (blue fluorescence). The scale bar is 50 μm. **B)** Quantitation of AB319 binding to transiently transfected HEK293 cells, as shown in A. Data points represent mean fluorescent intensities (MFI) measured from two 40x images per concentration and error bars indicate the standard deviation.
(TIF)

S3 Fig Live cell based assay with transiently transfected cells. HEK293 cells transiently expressing the α7 AChR subunit were stained with α-bungarotoxin or human serum (diluted 1:40), and then fixed and permeabilized. α7 AChR expression was visualized with AB319 (A and C) (1:800) and by goat-anti-rat IgG-Alexa594 (1:500). α-bungarotoxin binding was visualized in green (B). C) representative image of a co-staining of AB319 with IgG from serum from P4, a patient with psychosis that gave a weak positive result by RIA. The scale bar represents 50 μm. Cell nuclei are stained with DAPI (blue fluorescence).
(TIF)

S1 Table RIA results of complete cohort. (XLSX)

## References

[1] Hoffmann C, Zong S, Damas M, Molenaar P, Losen M, Martinez-Martinez P. Autoantibodies in Neuropsychiatric Disorders. antibodies. 2016;5(2). Epub 21 April 2016. 10.3390/antib5020009 10.3390/antib5020009 PMC6698850 31557990

[2] Freedman R, Adams CE, Leonard S. The alpha7-nicotinic acetylcholine receptor and the pathology of hippocampal interneurons in schizophrenia. J Chem Neuroanat. 2000;20(3–4):299–306. . 11207427 10.1016/s0891-0618(00)00109-5

[3] Gibbons A, Dean B. The Cholinergic System: An Emerging Drug Target for Schizophrenia. Curr Pharm Des. 2016;22(14):2124–33. . 26818859 10.2174/1381612822666160127114010

[4] De Luca V, Likhodi O, Van Tol HH, Kennedy JL, Wong AH. Regulation of alpha7-nicotinic receptor subunit and alpha7-like gene expression in the prefrontal cortex of patients with bipolar disorder and schizophrenia. Acta Psychiatr Scand. 2006;114(3):211–5. 10.1111/j.1600-0447.2006.00785.x . 16889592 10.1111/j.1600-0447.2006.00785.x

[5] Gass N, Weber-Fahr W, Sartorius A, Becker R, Didriksen M, Stensbol TB, et al An acetylcholine alpha7 positive allosteric modulator rescues a schizophrenia-associated brain endophenotype in the 15q13.3 microdeletion, encompassing CHRNA7. Eur Neuropsychopharmacol. 2016;26(7):1150–60. 10.1016/j.euroneuro.2016.03.013 . 27061851 10.1016/j.euroneuro.2016.03.013

[6] Liu X, Hong X, Chan RC, Kong F, Peng Z, Wan X, et al Association study of polymorphisms in the alpha 7 nicotinic acetylcholine receptor subunit and catechol-o-methyl transferase genes with sensory gating in first-episode schizophrenia. Psychiatry Res. 2013;209(3):431–8. 10.1016/j.psychres.2013.03.027 . 23598060 10.1016/j.psychres.2013.03.027

[7] Guan ZZ, Zhang X, Blennow K, Nordberg A. Decreased protein level of nicotinic receptor alpha7 subunit in the frontal cortex from schizophrenic brain. Neuroreport. 1999;10(8):1779–82. . 10501574 10.1097/00001756-199906030-00028

[8] Chandley MJ, Miller MN, Kwasigroch CN, Wilson TD, Miller BE. Increased antibodies for the alpha7 subunit of the nicotinic receptor in schizophrenia. Schizophr Res. 2009;109(1–3):98–101. 10.1016/j.schres.2009.01.023 . 19243919 10.1016/j.schres.2009.01.023

[9] Koval L, Lykhmus O, Kalashnyk O, Bachinskaya N, Kravtsova G, Soldatkina M, et al The presence and origin of autoantibodies against alpha4 and alpha7 nicotinic acetylcholine receptors in the human blood: possible relevance to Alzheimer's pathology. J Alzheimers Dis. 2011;25(4):747–61. 10.3233/JAD-2011-101845 . 21593571 10.3233/JAD-2011-101845

[10] Lykhmus O, Voytenko L, Koval L, Mykhalskiy S, Kholin V, Peschana K, et al alpha7 Nicotinic acetylcholine receptor-specific antibody induces inflammation and amyloid beta42 accumulation in the mouse brain to impair memory. PLoS One. 2015;10(3):e0122706 10.1371/journal.pone.0122706 ; PubMed Central PMCID: PMCPMC4376778. 25816313 10.1371/journal.pone.0122706 PMC4376778

[11] Kalkman HO, Feuerbach D. Modulatory effects of alpha7 nAChRs on the immune system and its relevance for CNS disorders. Cell Mol Life Sci. 2016;73(13):2511–30. 10.1007/s00018-016-2175-4 ; PubMed Central PMCID: PMCPMC4894934. 26979166 10.1007/s00018-016-2175-4 PMC4894934

[12] Lindstrom JM, Seybold ME, Lennon VA, Whittingham S, Duane DD. Antibody to acetylcholine receptor in myasthenia gravis. Prevalence, clinical correlates, and diagnostic value. Neurology. 1976;26(11):1054–9. . 988512 10.1212/wnl.26.11.1054

[13] Vincent A, Newsom-Davis J. Acetylcholine receptor antibody as a diagnostic test for myasthenia gravis: results in 153 validated cases and 2967 diagnostic assays. J Neurol Neurosurg Psychiatry. 1985;48(12):1246–52. ; PubMed Central PMCID: PMC1028609. 4087000 10.1136/jnnp.48.12.1246 PMC1028609

[14] Apiwattanakul M, McKeon A, Pittock SJ, Kryzer TJ, Lennon VA. Eliminating false-positive results in serum tests for neuromuscular autoimmunity. Muscle Nerve. 2010;41(5):702–4. 10.1002/mus.21653 . 20405502 10.1002/mus.21653

[15] Winston N, Vernino S. Recent advances in autoimmune autonomic ganglionopathy. Curr Opin Neurol. 2010;23(5):514–8. 10.1097/WCO.0b013e32833d4c7f . 20634694 10.1097/WCO.0b013e32833d4c7f

[16] Vernino S, Low PA, Fealey RD, Stewart JD, Farrugia G, Lennon VA. Autoantibodies to ganglionic acetylcholine receptors in autoimmune autonomic neuropathies. N Engl J Med. 2000;343(12):847–55. 10.1056/NEJM200009213431204 . 10995864 10.1056/NEJM200009213431204

[17] Paterson D, Nordberg A. Neuronal nicotinic receptors in the human brain. Progress in Neurobiology. 2000;61(1):75–111. 10.1016/S0301-0082(99)00045-3. 10759066

[18] Millar NS, Gotti C. Diversity of vertebrate nicotinic acetylcholine receptors. Neuropharmacology. 2009;56(1):237–46. 10.1016/j.neuropharm.2008.07.041 . 18723036 10.1016/j.neuropharm.2008.07.041

[19] Gotti C, Fornasari D, Clementi F. Human neuronal nicotinic receptors. Prog Neurobiol. 1997;53(2):199–237. Epub 1997/11/19. . 9364611 10.1016/s0301-0082(97)00034-8

[20] Court JA, Martin-Ruiz C, Graham A, Perry E. Nicotinic receptors in human brain: topography and pathology. Journal of Chemical Neuroanatomy. 2000;20(3–4):281–98. 10.1016/S0891-0618(00)00110-1. 11207426

[21] van Winkel R, van Os J, Celic I, Van Eyck D, Wampers M, Scheen A, et al Psychiatric diagnosis as an independent risk factor for metabolic disturbances: results from a comprehensive, naturalistic screening program. J Clin Psychiatry. 2008;69(8):1319–27. . 18681750 10.4088/jcp.v69n0817

[22] Pina-Camacho L, Diaz-Caneja CM, Saiz PA, Bobes J, Corripio I, Grasa E, et al Pharmacogenetic study of second-generation antipsychotic long-term treatment metabolic side effects (the SLiM Study): rationale, objectives, design and sample description. Rev Psiquiatr Salud Ment. 2014;7(4):166–78. 10.1016/j.rpsm.2014.05.004 . 25440735 10.1016/j.rpsm.2014.05.004

[23] Kuryatov A, Mukherjee J, Lindstrom J. Chemical chaperones exceed the chaperone effects of RIC-3 in promoting assembly of functional alpha7 AChRs. PLoS One. 2013;8(4):e62246 10.1371/journal.pone.0062246 PubMed Central PMCID: PMCPMC3634732. 23638015 10.1371/journal.pone.0062246 PMC3634732

[24] Gomez AM, Vrolix K, Martinez-Martinez P, Molenaar PC, Phernambucq M, van der Esch E, et al Proteasome inhibition with bortezomib depletes plasma cells and autoantibodies in experimental autoimmune myasthenia gravis. J Immunol. 2011;186(4):2503–13. 10.4049/jimmunol.1002539 . 21239719 10.4049/jimmunol.1002539

[25] de Witte LD, Hoffmann C, van Mierlo HC, Titulaer MJ, Kahn RS, Martinez-Martinez P, et al Absence of N-Methyl-D-Aspartate Receptor IgG Autoantibodies in Schizophrenia: The Importance of Cross-Validation Studies. JAMA Psychiatry. 2015;72(7):731–3. 10.1001/jamapsychiatry.2015.0526 . 25970159 10.1001/jamapsychiatry.2015.0526

[26] McKeon A, Pittock SJ, Lennon VA. CSF complements serum for evaluating paraneoplastic antibodies and NMO-IgG. Neurology. 2011;76(12):1108–10. 10.1212/WNL.0b013e318211c379 ; PubMed Central PMCID: PMCPMC3270330. 21422462 10.1212/WNL.0b013e318211c379 PMC3270330

[27] Gresa-Arribas N, Titulaer MJ, Torrents A, Aguilar E, McCracken L, Leypoldt F, et al Antibody titres at diagnosis and during follow-up of anti-NMDA receptor encephalitis: a retrospective study. Lancet Neurol. 2014;13(2):167–77. 10.1016/S1474-4422(13)70282-5 ; PubMed Central PMCID: PMC4006368. 24360484 10.1016/S1474-4422(13)70282-5 PMC4006368

[28] Vasic N, Connemann BJ, Wolf RC, Tumani H, Brettschneider J. Cerebrospinal fluid biomarker candidates of schizophrenia: where do we stand? Eur Arch Psychiatry Clin Neurosci. 2012;262(5):375–91. 10.1007/s00406-011-0280-9 . 22173848 10.1007/s00406-011-0280-9

[29] Sine SM. The nicotinic receptor ligand binding domain. J Neurobiol. 2002;53(4):431–46. 10.1002/neu.10139 . 12436411 10.1002/neu.10139

[30] Huang S, Li SX, Bren N, Cheng K, Gomoto R, Chen L, et al Complex between alpha-bungarotoxin and an alpha7 nicotinic receptor ligand-binding domain chimaera. Biochem J. 2013;454(2):303–10. 10.1042/BJ20130636 ; PubMed Central PMCID: PMCPMC3920732. 23800261 10.1042/BJ20130636 PMC3920732

[31] Anon (n.d.). Acetylcholine Receptor (Muscle AChR) binding antibody, serum. Mayo Medical Laboratories https://www.mayomedicallaboratories.com/test-catalog/Clinical+and+Interpretive/37427, accessed November 21, 2018.

[32] Sundar K, Venkatasubramanian S, Shanmugam S, Arthur P, Subbaraya R, Hazeena P. False positive immunoassay for acetyl choline receptor antibody (AChR Ab) in patients exposed to polyvalent antisnake venom. J Neuroimmunol. 2017;311:68–70. Epub 2017/08/24. 10.1016/j.jneuroim.2017.08.004 . 28830630 10.1016/j.jneuroim.2017.08.004

[33] Somnier FE. Clinical implementation of anti-acetylcholine receptor antibodies. J Neurol Neurosurg Psychiatry. 1993;56(5):496–504. Epub 1993/05/01. ; PubMed Central PMCID: PMCPMC1015008. 8505642 10.1136/jnnp.56.5.496 PMC1015008

[34] Howard FM Jr., Lennon VA, Finley J, Matsumoto J, Elveback LR. Clinical correlations of antibodies that bind, block, or modulate human acetylcholine receptors in myasthenia gravis. Ann N Y Acad Sci. 1987;505:526–38. Epub 1987/01/01. . 3479935 10.1111/j.1749-6632.1987.tb51321.x

