# Supplementary figures and images for: Alpha7 acetylcholine receptor autoantibodies are rare in sera of patients diagnosed with schizophrenia or bipolar disorder
Source: PLoS One. 2018 Dec 6;13(12):e0208412. doi: 10.1371/journal.pone.0208412 (PMC6283580; doi:10.1371/journal.pone.0208412)

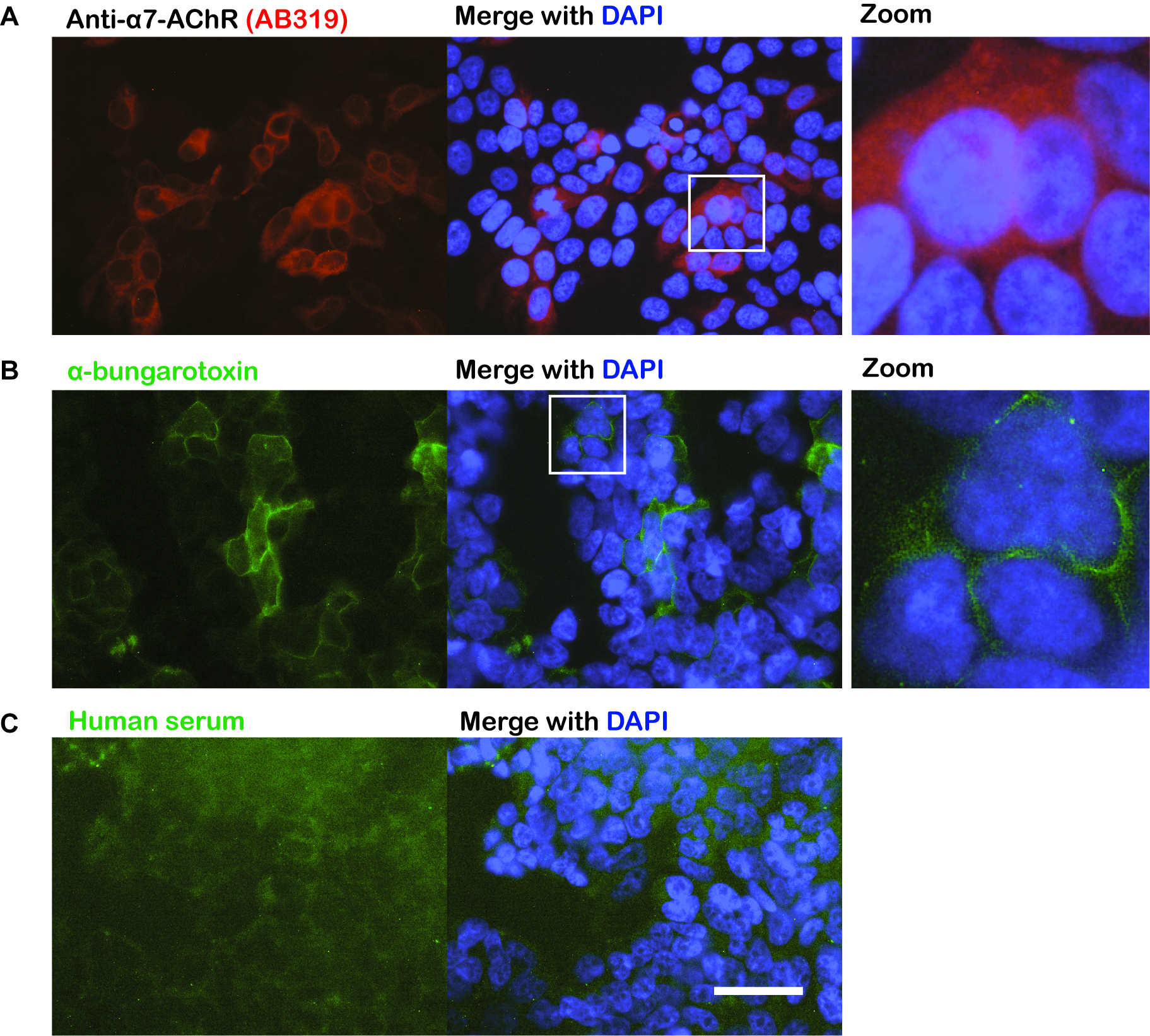

Supplement: S1 Fig — Cells stably expressing α7 AChR were cultured with chemical chaperones. α7 AChR expression was detected by fixed CBA with AB319 followed by secondary goat-anti-rat-Alexa594 (A, red fluorescence) or by live CBA with Alexa488-labelled α-bungarotoxin (B, green fluorescence). C) represents a staining with serum (diluted 1:40) from a healthy control on live CBA. Bound antibodies were detected by goat-anti-human-IgG Fcγ-Alexa488. The scale bar represents 50 μm. Cell nuclei are stained with DAPI (blue fluorescence). (TIF) [file pone.0208412.s001.tif]

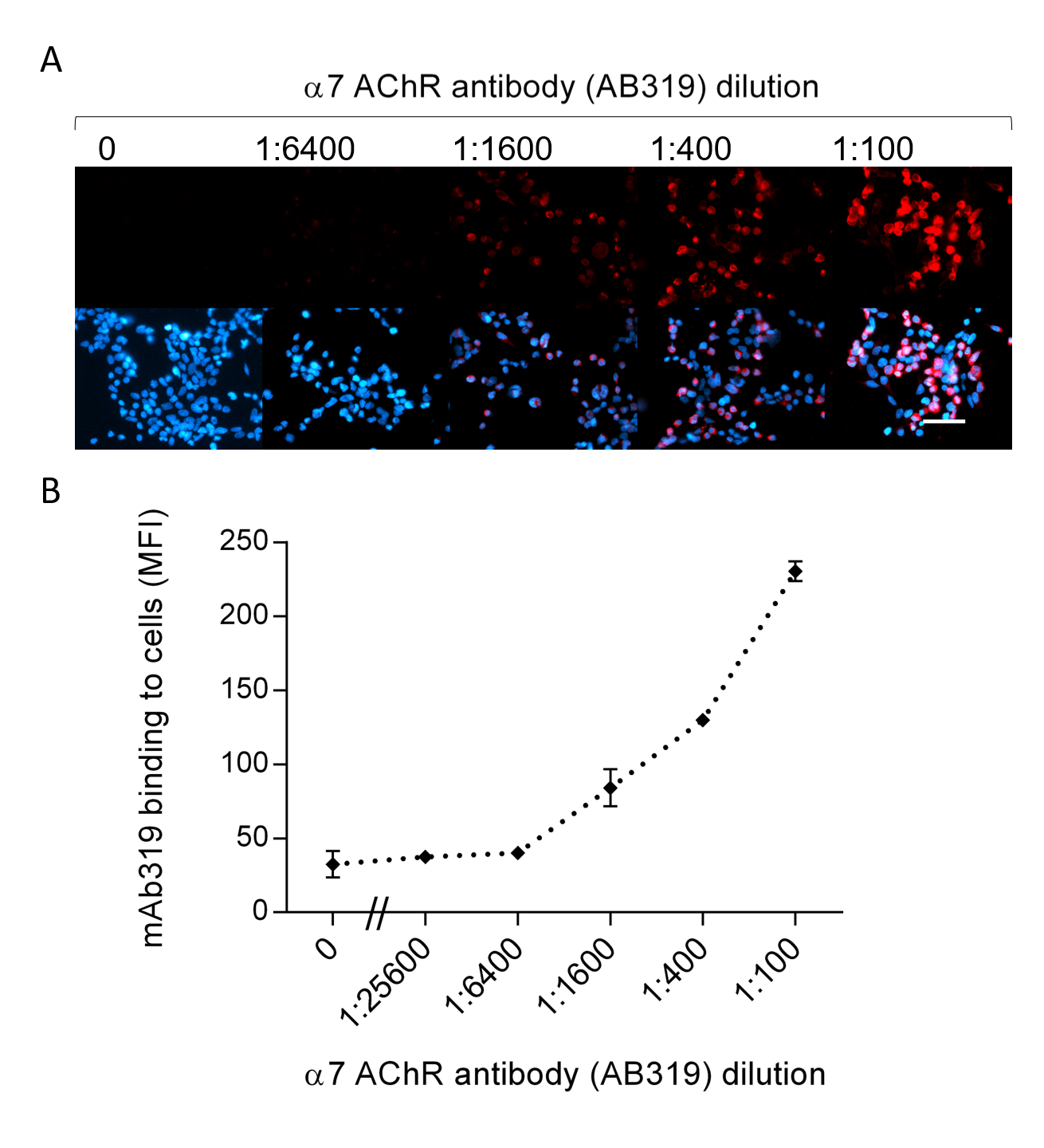

Supplement: S2 Fig — A) AB319 (at dilutions as indicated above the images) was used to stain paraformaldehyde-fixed, Triton-X100-permeabilized, α7 AChR-expressing HEK293 cells after transient transfection. AB319-binding was detected by goat-anti-rat IgG-Alexa594 (1:500; red fluorescence). Cell nuclei are stained with DAPI (blue fluorescence). The scale bar is 50 μm. B) Quantitation of AB319 binding to transiently transfected HEK293 cells, as shown in A. Data points represent mean fluorescent intensities (MFI) measured from two 40x images per concentration and error bars indicate the standard deviation. (TIF) [file pone.0208412.s002.tif]

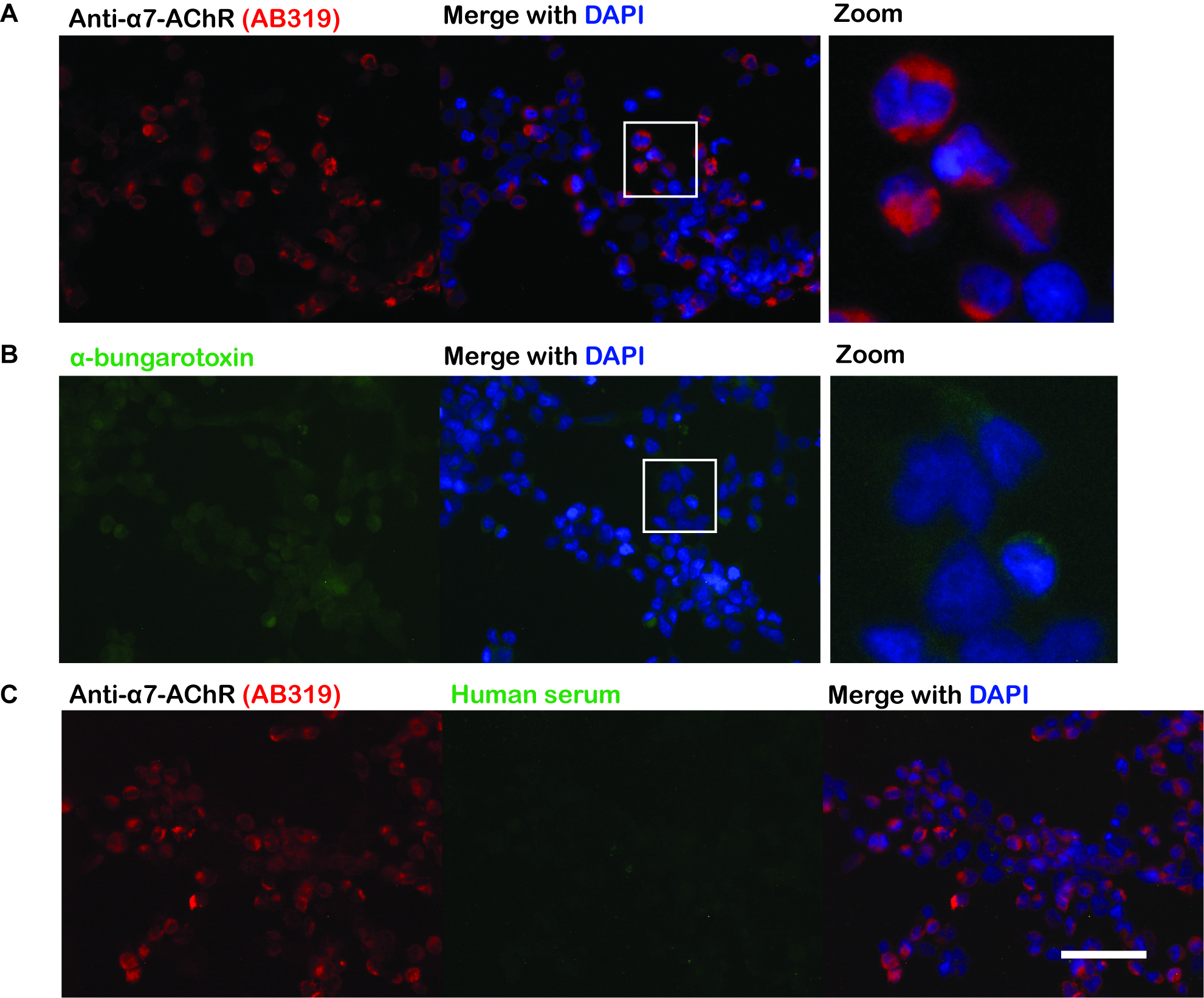

Supplement: S3 Fig — HEK293 cells transiently expressing the α7 AChR subunit were stained with α-bungarotoxin or human serum (diluted 1:40), and then fixed and permeabilized. α7 AChR expression was visualized with AB319 (A and C) (1:800) and by goat-anti-rat IgG-Alexa594 (1:500). α-bungarotoxin binding was visualized in green (B). C) representative image of a co-staining of AB319 with IgG from serum from P4, a patient with psychosis that gave a weak positive result by RIA. The scale bar represents 50 μm. Cell nuclei are stained with DAPI (blue fluorescence). (TIF) [file pone.0208412.s003.tif]
